# Unusual Presentation of Hypopituitarism Caused by Internal Carotid Artery Aneurysm

**DOI:** 10.7759/cureus.95284

**Published:** 2025-10-24

**Authors:** Shria Datta, Shyama Shyama, Surya N Prasad

**Affiliations:** 1 General Medicine, All India Institute of Medical Sciences Patna, Patna, IND; 2 Radiodiagnosis, All India Institute of Medical Sciences Patna, Patna, IND

**Keywords:** endovascular coiling, hypopituitarism, internal carotid artery aneurysm, intracranial aneurysm, panhypopituitarism, sheehan syndrome

## Abstract

Hypopituitarism is most commonly caused by pituitary or parasellar tumors, while vascular lesions such as intracranial aneurysms are exceedingly rare etiologies. We report a unique case of a 42-year-old woman with a long-standing history of hypothyroidism who presented with generalized edema, hypotension, amenorrhea, and recurrent hypoglycemia. Biochemical assessment revealed panhypopituitarism-secondary hypothyroidism [free thyroxine (FT4) <0.88 pmol/L, thyroid-stimulating hormone (TSH) 3.36 µIU/mL] and secondary adrenal insufficiency, with extremely low serum cortisol (<0.16 µg/dL) and adrenocorticotropic hormone (ACTH) 9 pg/mL. Gonadotropins were also reduced [luteinizing hormone (LH) 1.49 mIU/mL, follicle-stimulating hormone (FSH) 5.12 mIU/mL].

Magnetic resonance imaging (MRI) of the brain demonstrated an empty sella with a left supraclinoid internal carotid artery (ICA) saccular aneurysm extending into the suprasellar region. Digital subtraction angiography confirmed the diagnosis, and the patient underwent successful endovascular coiling. Following the intervention, her electrolyte abnormalities resolved, and she remained stable on hydrocortisone and thyroxine supplementation. This case highlights the importance of considering ICA aneurysm in the differential diagnosis of hypopituitarism, as its presentation can mimic pituitary adenoma. In contrast to prior reports where visual disturbances predominated, this case presents isolated endocrine dysfunction without visual or neurological symptoms. Additionally, the coexistence of an empty sella and supraclinoid ICA aneurysm-induced panhypopituitarism is exceptionally rare. Early recognition with neuroimaging and timely intervention are crucial to prevent life-threatening endocrine crises and neurological complications.

## Introduction

Hypopituitarism is a consequence of the pituitary gland failing to produce either completely or partially one or more of its hormones. In case of partial hormone deficiency, the most commonly affected hormones are growth hormone (GH) and the gonadotropins, luteinizing hormone (LH) and follicle-stimulating hormone (FSH), followed by deficiencies in thyroid-stimulating hormone (TSH), adrenocorticotropic hormone (ACTH), and prolactin [1].

A supraclinoid internal carotid artery (ICA) aneurysm is a rare condition. Intracranial aneurysms are fairly common and characteristically asymptomatic, often discovered by chance. However, they have the potential to occasionally rupture, resulting in a subarachnoid hemorrhage [2]. Mass effects may be caused by a larger aneurysm. Supraclinoid aneurysms are intradural aneurysms that form on the ICA at its origin beyond the distal dural ring, extending to the carotid terminus. It is most often caused by a mass in the sellar or parasellar region, typically a tumor. Magnetic resonance imaging (MRI) is regarded as an effective imaging technique for making differential diagnosis.

Intrasellar aneurysm is a rare etiology of hypopituitarism accounting for approximately 0.17% of all reported cases [3]. Hormonal deficiency may result from mechanical compression and pulsatile effects of the aneurysm, as well as impairment of the pituitary microvascular supply.

We report a rare case involving a 42-year-old woman who exhibited features of panhypopituitarism diagnosed with an unruptured supraclinoid ICA aneurysm with suprasellar extension. In this case, the coexistence of an empty sella with an aneurysm adds to the diagnostic challenge [4] and successful endovascular coiling highlights the importance of early recognition and intervention.

## Case presentation

A female patient, aged 42, with a 15-year background of hypothyroidism maintained on thyroxine, was admitted to the internal medicine ward with the chief complaints of swelling over the face and bilateral feet for 15 days. The patient was apparently well when she developed swelling over the face and bilateral feet, which was acute in onset and gradually progressive. The swelling started from the face and gradually progressed to both feet, followed by both hands. She was hypotensive, and her physical examination indicated edema in the face, bilateral feet, and hands. Her pulse rate and temperature were normal. Bilateral vesicular breath sounds along with bilateral fine crepts were heard on auscultation. There were no abnormal findings in her cardiovascular, neurological, and abdominal examination.

The patient reported her last menstrual period to be 10 years ago. Her obstetrical history of two gestations included two live births by normal vaginal delivery. The postpartum period after the birth of her first child was complicated by excessive blood loss, requiring one unit of blood transfusion. Lactational failure after both deliveries was noted. She had episodes of hypoglycemia, and investigations revealed hyponatremia with a sodium value of 119 meq/L and serum potassium value of 4.5 meq/L.

Secondary hypothyroidism was noted with TSH at 3.3 micro IU/ml and FT4 less than 0.8 pmol/L. Serum cortisol was less than 0.16 ug/dl, and ACTH level was 9 pg/ml. Levels of LH were 1.49 mlU/ml and FSH was 5.12 mlU/ml, as given in Table 1. The patient was thus diagnosed as a case of panhypopituitarism. Hydrocortisone and thyroxine supplementation were started.

**Table 1:** Laboratory Investigation LH: luteinizing hormone; FSH: follicle-stimulating hormone; TSH: thyroid-stimulating hormone; FT3: free triiodothyronine; FT4: free thyroxine; eE2: enhanced estradiol; ACTH: adrenocorticotropic hormone; WBC: white blood cells; RBC: red blood cells; PCV: packed cell volume; MCV: mean corpuscular volume; MCH: mean corpuscular hemoglobin; MCHC: mean corpuscular haemoglobin concentration; RDW CV: red cell distribution width coefficient of variation.

Investigation Name	Result	Unit	Reference Range
Biochemistry			
LH	1.49	mlU/ml	Female: Follicular Phase: 1.9 – 12.5 Mid cycle: 8.7 – 76.3 Luteal Phase: 0.5 – 16.9 Post menopausal: 15.9 – 54.0 Male: 1.5 – 9.3
FSH	5.12	mlU/ml	Male: 1.4 – 18.1 Female: Follicular Phase: 2.5 – 10.2 Mid cycle: 3.4 – 33.4 Luteal Phase: 1.5 – 9.1 Post menopausal: 23 – 116.3
Sodium	126.83	meq/l	135 - 145
Potassium	3.56	meq/l	3.5 - 5
Chloride	93.48	meq/l	98 - 107
Serum Cortisol	Less than 0.16	Ug/dl	4.30 -22
FT3	1.28	pmol/L	3.5 – 6.5
FT4	Less than 0.88	pmol/L	11.5 – 22.7
TSH	3.367	Micro IU/ml	0.35 – 5.5
Vitamin B12	391	pg/ml	211 - 911
Serum Folate	20.50	ng/ml	>5.38
eE2(Estradiol)	1.14	pg/ml	22.4 - 115
ACTH	9	pg/ml	9 - 52
Complete Haemogram			
Haemoglobin	9.1		12 – 15g/dL
WBC	4.04		4 – 10 Thousand/MicroLtr
Platelet Count	198		150 – 450 Thousand/MicroLtr
RBC Count	3.15		3.8 – 4.8 Million/MicroL
PCV	28.6		36.0 – 46.0 %
MCV	90.8		83 – 101 fL
MCH	28.9		27 – 32 pg
MCHC	31.8		31.5 – 34.5 g/dL
RDW CV	15.4		11.6 – 14 %
Neutrophils	81.8		40 – 80 %
Lymphocytes	15.8		20 – 40 %
Monocytes	2.2		2 – 10 %
Eosinophils	0.0		1 – 6 %
Basophils	0.2		0 – 1 %
Mean Platelet Volume	11.6		9.4 – 12.3 fL
Platelet Distribution Width	13.5		10.0 – 17.9 %
Other			

**Figure 1:**
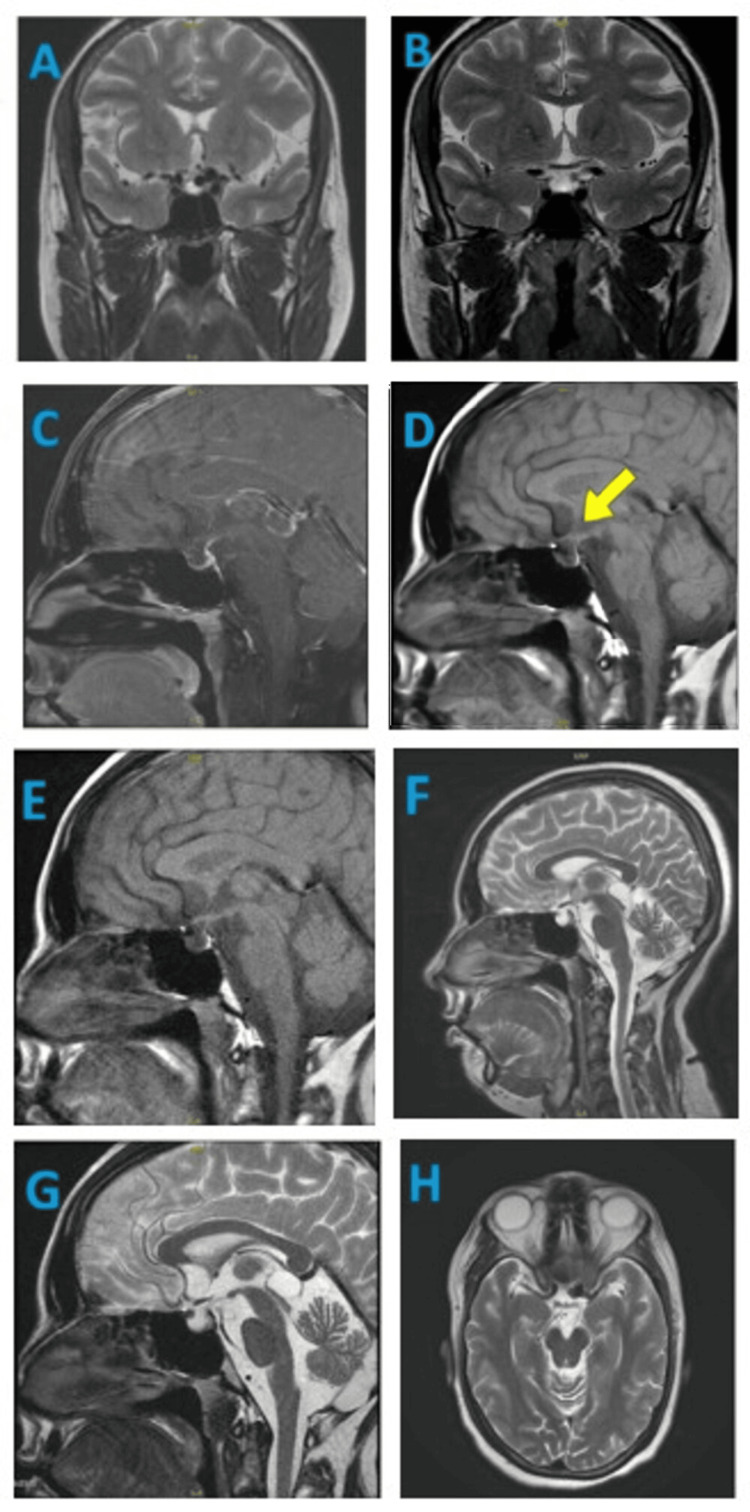
Magnetic resonance imaging of brain. Panels A–H: MRI of the brain demonstrating the sellar, suprasellar, and parasellar regions in multiple planes and sequences. Panels A and B: Coronal T2-weighted images delineate the pituitary gland, cavernous sinuses, optic chiasm, and adjacent temporal lobes. Panels C–E: Sagittal T1-weighted and contrast-enhanced images highlight the pituitary gland, infundibulum, optic chiasm, sphenoid sinus, and clivus. Panels F and G: Sagittal T2-weighted images demonstrate midline neuroanatomical structures, including the corpus callosum, third and fourth ventricles, brainstem, cerebellum, and pituitary gland. Panel H: Axial T2-weighted imaging provides visualization of the sellar and parasellar region, including the pituitary gland, cavernous sinuses, optic nerves, and temporal lobes.

MRI brain with pituitary protocol was done, which was suggestive of an empty sella turcica and a small pituitary gland with a left supraclinoid ICA saccular aneurysm, as given in Table 2 and Figure 1. A non-contrast computed tomography (CT) of the head showed a hyperdense foreign body with metallic beam hardening artifact in the left basifrontal region - likely post-coiling changes in a known case of left subarachnoid hemorrhage.

**Table 2:** MRI Brain-Plain and Contrast; Pituitary Protocol ICA: internal carotid artery.

MRI Brain-Plain and Contrast; Pituitary Protocol
Clinical Features: Euvolemic hyponatremia with secondary hyperthyroidism.
Findings:
Empty sella turcica with maximum pituitary thickness measuring 1.5mm noted. A normal posterior pituitary bright spot is seen.
There is evidence of a medially directed saccular aneurysm measuring ~ 6 x 8 mm arising from the supraclinoid segment of the left ICA, extending into the suprasellar cistern, maintaining fat planes with the pituitary stalk is noted.
Note is made of a cystic lesion of size 14.3 mm x 13.5 mm in the pineal gland. Smooth rim enhancement is noted on post contrast study – Pineal gland cyst.
Impression:
Empty sella turcica and small pituitary gland.
A left supraclinoid ICA saccular aneurysm. Digital Subtraction Angiography (DSA) evaluation advised.
Pineal gland cyst.

Diagnostic digital subtraction angiography (DSA) showed mild dysplastic changes in the left supraclinoid ICA with an ICA - superior hypophyseal artery (SHA) lobulated aneurysm and a small right paraophthalmic ICA aneurysm. DSA further revealed a lobulated, medially directed, wide-neck ICA - SHA aneurysm and a prominent infundibulum. Control DSA showed near-complete embolization of the aneurysm with residual filling in the neck region. A residual aneurysm and another smaller aneurysm arising at the right SHA origin are planned to be coil embolized in another session, as given in Table 3.

**Table 3:** Cerebral Digital Subtraction Angiography ICA: internal carotid artery; SHA: superior hypophyseal artery.

Cerebral Digital Subtraction Angiography
Indications: Headache, hypopituitarism, magnetic resonance imaging (MRI) suggestive of left ICA – SHA aneurysm.
Findings:
Left ICA Injection: Supraclenoid segment appears mildly dysplastic. A medially directed lobulated aneurysm (8.2mmX5mm) is seen arising at the origin of the SHA.
Right ICA Injection: A small (3.5 cm X 2.7mm) Inferomedially directed paraophtamic ICA aneurysm is seen.
Impression:
Mildly dysplastic left supraclenoid ICA with an ICA - SHA lobulated aneurysm.
Small right paraophtalmic ICA aneurysm.

The arterial sheath was removed, and the puncture wound was closed with a vascular closure device. No periprocedural complications occurred. Therapeutic coiling followed by extubation was done under general anesthesia, and the patient was kept in the medical intensive care unit (MICU) until extubation. No further hypoglycemic episodes were noted. Her serum sodium levels normalized, and the patient was discharged on hydrocortisone and thyroxine supplementation. The investigation results can be seen in the tables below.

## Discussion

Unruptured intracranial aneurysms are most frequently found at arterial bifurcations within the circle of Willis and are estimated to occur in about 3% of the population. They typically remain asymptomatic until rupture, which can lead to subarachnoid hemorrhage, which is a complication associated with significant morbidity and mortality [5]. An intrasellar aneurysm represents an uncommon etiology of hypopituitarism, reported in roughly 0.17% of cases.

A systematic review analyzing 40 documented cases of intrasellar aneurysms revealed that the most common clinical manifestations of ICA aneurysms were headache and reduced visual acuity, each occurring in 61% of patients. Endocrinopathies accounted for 57% of cases, while 21% experienced symptomatic hyponatremia. Additionally, cranial nerve palsies not involving the optic nerve were reported in 18% of cases. The predominant endocrine alterations associated with sellar aneurysms included hyperprolactinemia (90%), ACTH deficiency (70%), TSH deficiency (60%), and gonadotropin deficiency (82%) [4]. These findings indicate that intrasellar aneurysms can lead to endocrine dysfunctions resembling those produced by pituitary macroadenomas. Thus, in previous reports, the predominant features were neurological and visual disturbances with endocrine dysfunction occurring as an associated finding.

In contrast, our patient had features of hypopituitarism-secondary hypothyroidism, adrenal insufficiency, and gonadotropin deficiency, with no cranial nerve or visual involvement. The likely explanation is compression of the surrounding pituitary parenchyma by the left supraclinoid ICA aneurysm with suprasellar extension, resulting in panhypopituitarism.

Previous case reports had patients who presented with headache, blurred vision, and adrenal insufficiency due to ICA aneurysms, indicating that the mechanisms could include either compression of the hypothalamus disrupting releasing factors or a direct destructive effect of the aneurysm on pituitary tissue [6]. A report of another patient described a 72-year-old male with persistent hyponatremia and hypoglycemia caused by an unruptured ophthalmic ICA aneurysm with sella extension [3]. Similarly, a 43-year-old female had a long history of amenorrhea owing to an ICA aneurysm compressing the pituitary gland [7].

Compared to these cases, this patient’s history of ten years of amenorrhea, accompanied by serum follicle-stimulating hormone (FSH) and luteinizing hormone (LH) levels lower than expected for the postmenopausal state, suggests that early menopause was the result of pituitary dysfunction. In this case, hypothyroidism due to impaired TSH secretion by the anterior pituitary gland serves as the major factor underlying the patient's clinical manifestations. The abnormal thyroid profile accounts for the cause of fatigue, decreased appetite, swelling, hair thinning, and dry, wrinkled skin. The patient's initial presentation of hypotension, tachycardia, and altered sensorium was indicative of an adrenal crisis. This was attributed to ACTH insufficiency, as evidenced by decreased serum levels of both ACTH and cortisol. This was contrary to most previous reports, where visual symptoms dominated; the absence of neuro-ophthalmic signs in this case emphasizes an uncommon clinical trajectory.

A large cohort study at Mayo Clinic, involving over 4,000 patients with hypopituitarism, revealed that sellar aneurysms accounted for less than 0.2% of pituitary dysfunction cases [8]. To date, there have been only two documented instances of pituitary dysfunction resulting from an unruptured ICA supraclinoid segment aneurysm [3].

Aneurysms located in the supraclinoid segment of the ICA that are smaller than 10 mm in diameter frequently lack clinical manifestations. Aneurysms may progressively enlarge and manifest with symptoms such as headache and cranial nerve palsies, most commonly visual dysfunction due to compressive effect on the anterior optic pathway. Additionally, anterior optic pathway ischemia may result from pressure exerted on the ophthalmic artery. MRI is commonly used to detect unruptured supraclinoid carotid aneurysms, particularly when patients exhibit clinical phenomena resulting from visual pathway compression. MRI serves both to exclude other diagnostic possibilities and guide ongoing assessment. A definitive method for confirming this diagnosis is digital subtraction angiography.

Endovascular coiling was necessary for our patient due to the significant endocrine compromise. The intervention successfully stabilized her biochemical profile and prevented further adrenal crises, highlighting the importance of timely recognition and treatment [9].

## Conclusions

ICA aneurysm with stellar and suprasellar extensions can be a cause of severe compressive symptoms such as visual dysfunction and endocrinopathies, mimicking pituitary adenoma, as symptoms resemble hypopituitarism. This case highlights the clinical significance of maintaining a high index of suspicion for vascular lesions in patients presenting with unexplained endocrine deficiencies, particularly when visual symptoms are absent.

MRI with a dedicated pituitary protocol, together with vascular imaging such as MR angiography or digital subtraction angiography, is critical in reliably distinguishing ICA aneurysms from pituitary tumors. Early detection avoids potentially disastrous complications resulting from manipulation of such aneurysms during transsphenoidal or other pituitary operations. Preoperative intracranial vasculature evaluation should thus be a standard consideration in sellar or parasellar mass patients.

Reassessment of the hypothalamic-pituitary axis is necessary after endoscopic treatment of an ICA aneurysm in order to determine recovery or persistence of pituitary hormone deficits and monitor treatment outcomes. This case highlights the need for early neuroimaging, multidisciplinary collaboration between radiology, neurosurgery, and endocrinology, and ongoing hormonal monitoring to ensure comprehensive care and prevent life-threatening endocrine crises.

## References

[1] Diri H, Tanriverdi F, Karaca Z (2014). Extensive investigation of 114 patients with Sheehan's syndrome: a continuing disorder. Eur J Endocrinol.

[2] Khattab DI, Aljeradat B, Batarseh DR, Al-Abadi H, Shehadeh M (2024). Bilateral giant supraclinoid carotid aneurysms causing obstructive hydrocephalus: case report and literature review. Cureus.

[3] Ičin T, Stepanović K, Bajkin I (2024). An unusual presentation of hypopituitarism caused by a sellar aneurysm. Arch Endocrinol Metab.

[4] Hanak BW, Zada G, Nayar VV, Thiex R, Du R, Day AL, Laws ER (2012). Cerebral aneurysms with intrasellar extension: a systematic review of clinical, anatomical, and treatment characteristics. J Neurosurg.

[5] Williams LN, Brown RD Jr (2013). Management of unruptured intracranial aneurysms. Neurol Clin Pract.

[6] Mulpuri N, Ghayee HK, Abramowitz J, Mirfakhraee S (2023). Internal carotid artery aneurysm disguised as pituitary macroadenoma. JCEM Case Rep.

[7] Oikawa N, Misaki K, Aono D, Nambu I, Hayashi Y, Uchiyama N, Nakada M (2022). Panhypopituitarism caused by an unruptured giant cavernous internal carotid artery aneurysm compressing the pituitary gland treated with a flow-diverting stent: a case report. Surg Neurol Int.

[8] Heshmati HM, Fatourechi V, Dagam SA, Piepgras DG (2001). Hypopituitarism caused by intrasellar aneurysms. Mayo Clinic Proceedings.

[9] Badrawi N, Iqbal SS, Ahmed A, Iqbal SS (2022). Unruptured bilateral supra-clinoid internal carotid artery aneurysms: a case report. Radiol Case Rep.

